# Elevated Free Thyroxine Levels Might Alter the Effect of the Lipid Profile on Insulin Resistance in Type 2 Diabetes Mellitus

**DOI:** 10.3390/diagnostics13162656

**Published:** 2023-08-11

**Authors:** Yi-Wei Lee, Ting-Teng Yang, Yan-Yu Lin, Yu-Shan Hsieh

**Affiliations:** 1 Department of Internal Medicine, Taipei Medical University Hospital, Taipei City 11030, Taiwan; 2 Division of Endocrinology and Metabolism, Department of Internal Medicine, Taipei Medical University Hospital, Taipei City 11030, Taiwan; 3 School of Nursing, National Taipei University of Nursing and Health Sciences, Taipei City 11230, Taiwan; 4 Department of Research, Taipei Medical University Hospital, Taipei City 11030, Taiwan

**Keywords:** type 2 diabetes mellitus, hyperthyroidism, insulin resistance, lipid profile, thyroid hormone

## Abstract

Previous studies have shown that hyperthyroidism is associated with heightened insulin resistance and dyslipidemia. Therefore, in this study, we aim to explore the relationship between elevated thyroid hormone levels and the lipid profile in insulin resistance in patients with type 2 diabetes mellitus (T2DM) with hyperthyroidism. A total of 177 participants were included and grouped according to diagnosis. The serum test results demonstrated that free thyroxine (FT4) increased the insulin resistance index (HOMA-IR) by positively correlating with triglyceride (TG) levels (*p* = 0.005, r^2^ = 0.35). In patients with T2DM with hyperthyroidism, the decreasing high-density lipoprotein levels showed an association with HOMA-IR (*p* = 0.005). Among all the patients, with different levels of FT4, the areas under the ROC curve (AUCs) of the TG level, TG/high-density lipoprotein ratio, and HOMA-IR were 0.620 (95% CI: 0.536 to 0.698), 0.614 (95% CI: 0.530 to 0.692), and 0.722 (95% CI: 0.645 to 0.791), respectively. Our results suggest that elevated FT4 levels due to hyperthyroidism could alter the association with the lipid profile and insulin resistance in patients with T2DM. We also suggest that among all the included patients with T2DM, irrespective of the presence of hyperthyroidism, FT4 levels are positively correlated with insulin resistance.

## 1. Introduction

Insulin resistance is usually linked with dyslipidemia, a pathophysiologic factor associated with type 2 diabetes mellitus (T2DM). Additionally, several studies have described the association of oxidative stress [1], inflammation [2], hyperinsulinemia [3], and lipid metabolic disease [4] with insulin resistance in T2DM. Previous studies have identified three primary factors related to dyslipidemia in insulin resistance: increased triglyceride (TG) levels, decreased high-density lipoprotein (HDL) cholesterol, and changes in the composition of low-density lipoprotein (LDL) cholesterol [5]. Moreover, a recent study demonstrated that the TG/HDL ratio was positively associated with insulin resistance [6]. It could be seen that lipid metabolism is closely related to insulin resistance.

The prevalence of thyroid disorders in diabetic populations is 13.4%, and it is believed that patients with T2DM are more prone to thyroid disorders [7]. It is well known that abnormal thyroid function can lead to clinical alterations such as energy expenditure, glucose metabolism, and lipid metabolism [8]. Moreover, free thyroxine (FT4) has been reported to regulate both insulin agonistic and antagonistic reactions in different tissues or organs [9]. Serum FT4 levels have also been reported to be negatively correlated with TG levels and positively correlated with HDL cholesterol levels [10]. Hyperthyroidism has also been shown to be associated with increased glucose intolerance and heightened insulin resistance. Therefore, higher levels of thyroid hormone may increase the rate of liver gluconeogenesis and glycogenolysis [11].

Therefore, in patients with T2DM complicated with hyperthyroidism, it is important to determine whether the effect of the lipid profile on insulin resistance is altered compared to that in patients without thyroid dysfunction. To this end, we included both clinical participants and those taken from a clinical database and classified them into a T2DM group and T2DM combined with hyperthyroidism group according to diagnosis to observe the effects of high thyroid hormone levels. We then used the homeostasis model assessment of insulin resistance index (HOMA-IR), a simple and effective method for evaluating insulin sensitivity, to evaluate the level of insulin resistance.

Observing changes in the lipid profile and determining whether high thyroid hormone levels alter the factors affecting insulin resistance can be used as early controls when encountering such patients in the future to achieve predictive and disease control effects.

## 2. Materials and Methods

### 2.1. Patients

Data from 179 patients who had received a diagnosis of T2DM with or without hyperthyroidism who were treated at the Taipei Medical University Hospital between December 2021 and December 2022 were retrospectively analyzed. The diagnosis was based on the International Classification of Diseases, Tenth Revision, Clinical Modification (ICD-10-CM) codes E05 and E11. The data derived from the Taipei Medical University Clinical Research Database (TMUCRD) were pseudo-anonymized. The requirement for patient consent was waived by the TMUCRD and the Institutional Review Board of Taipei Medical University (TMU-JIRB-N202209081). Additionally, 112 patients who had received a diagnosis of T2DM with or without hyperthyroidism who were treated at the Taipei Medical University Hospital between January 2021 and December 2022 were retrospectively analyzed. Patients were included in the study if they had received a diagnosis of T2DM with or without hyperthyroidism and had received continuous treatment between December 2021 and December 2022. The study was approved by the TMUCRD and the Institutional Review Board of Taipei Medical University (TMU-JIRB-N202107021). All clinical participants provided informed consent.

Women who were pregnant, those <20 years, and patients with neoplasms (ICD-10-CM C00-D49), complications, ill-defined cardiac disease or heart failure (ICD-10-CM I50-I99), and chronic liver disease or hepatitis (ICD-10-CM K70-K77) were excluded from the study. To exclude alcohol-related disease, we confirmed that the included patients did not have any alcohol-related diagnoses (ICD-10-CM F01-F99; K29.2; K85.2; K86.0). The sample size was verified using the G*Power software version 3.1.1. Calculations showed that with the study design of a case–control study and alpha error of 0.5, a total sample size of 176 can give 95% power with an effect size of 0.5.

Finally, 79 patients were included in the T2DM with hyperthyroidism group (T2DM/HTH group), and 98 patients were included in the T2DM group (T2DM group), resulting in a total of 177 participants.

### 2.2. Hematological Analysis

Blood samples were collected from participants after an 8 h fast. Hematological analyses, including measurement of the levels of serum lactate dehydrogenase (LDH), glucose (fasting blood glucose), glycosylated hemoglobin (HbA1c), insulin, free thyroxine (FT4), and thyroid-stimulating hormone (TSH), were performed. Glucose levels were analyzed using the hexokinase method, and HbA1c, LDH, insulin, FT4, and TSH levels were determined through high-performance liquid chromatography using an automatic analyzer (ADVIA Chemistry XPT, Erlangen, Germany).

### 2.3. HOMA-IR

The HOMA-IR is a simple and effective method for evaluating insulin sensitivity, which was calculated using the following formula: [glucose (mg/dL) × insulin (mIU/L)]/405.

### 2.4. Statistical Analysis

Statistical analyses were conducted using IBM SPSS Statistics version 22 (IBM Corporation, Somers, New York, NY, USA) and Med Calc 20.113 (Med Calc Software Ltd., Ostend, Belgium). Continuous variables are expressed as means with standard deviations (SD) or medians. Participants were categorized into T2DM+HTH and T2DM groups according to their diagnosis (ICD-10-CM E11. × with or without E05. ×). Baseline data were analyzed using independent t-tests for normally distributed variables and Chi-square tests ordinary trend tests for nonnormally distributed variables. Baseline variables were compared using Student’s *t*-test. The correlation was analyzed using Spearman tests.

To examine whether hyperthyroidism and the levels of FT4 and TSH were associated with HOMA-IR or HOMA-IR-related factors (HDL, TG, TG/HDL ratio), we plotted the receiver operating characteristic (ROC) curve and calculated the area under curve (AUC) values to assess their predictive ability in our study population. Additionally, linear regression analysis was performed to demonstrate the predictors of serum FT4 and TSH levels. Statistical significance was indicated at *p* < 0.05. All reported *p*-values are two-sided.

## 3. Results

### 3.1. Clinical Characteristics

**Figure 1:**
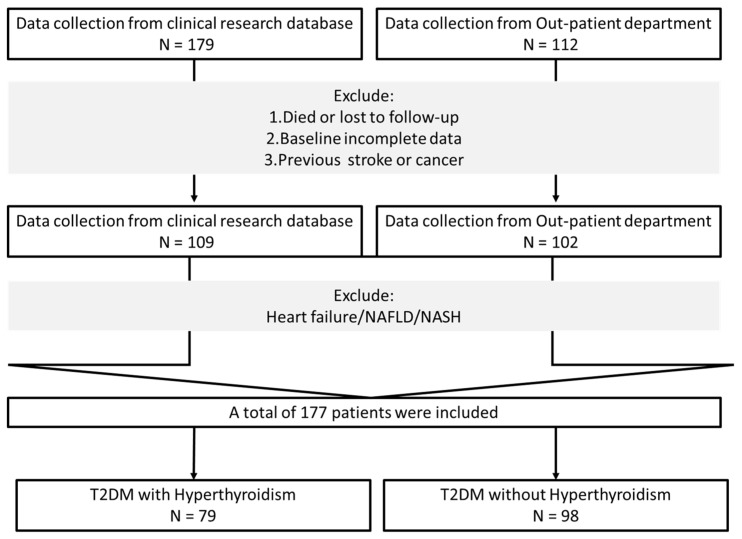
Flow chart for inclusion. NAFLD/NASH: Non-alcoholic fatty liver disease/nonalcoholic steatohepatitis, T2DM: Type 2 diabetes mellitus, N: number of participants.

A total of 177 patients with T2DM from Taipei Medical University Hospital from December 2021 to December 2022 were included. The selection process is summarized in Figure 1. The baseline demographic and clinical characteristics are described in Table 1. Among all of the participants, 72% were men, and the mean age was 53.1 (median: 53) years. The two groups of patients showed no significant differences in age, sex, or the presence of hypertension, dyslipidemia, cancer, stroke, heart failure, and non-alcoholic fatty liver disease/steatohepatitis (NAFLD/NASH). Furthermore, no significant difference was found in the use of drugs between the two groups.

### 3.2. Comparison of Insulin Resistance-Related Variables between Groups

The insulin resistance-related variables, including potential risk factors (HOMA-IR, insulin, glucose, HbA1C, LDH, TG, HDL cholesterol, LDL cholesterol, TG/HDL ratio, TC, FT4, and TSH), are presented in Table 1. In the T2DM+HTH group, a higher HOMA-IR was significantly associated with a doubling of serum glucose and insulin levels (*p* = 0.001). To further elucidate the causative factors of the higher HOMA-IR in the T2DM+HTH group, we compared the mean of the HbA1C, LDH, TG, HDL cholesterol, LDL cholesterol, TG/HDL ratio, TC, FT4, and TSH levels between the two groups. As a result, the patients in the T2DM+HTH group showed significantly higher TG (*p* = 0.047) and HDL (*p* = 0.019) levels.

### 3.3. Correlation of Serum Insulin Resistance with Related Factors in Each Group

As shown in Table 2, we observed significant differences in insulin resistance-related factors between the two groups. In the T2DM group (Table 2A), HOMA-IR was associated with the TG level (correlation coefficient: 0.25, *p* = 0.014) and TG/HDL ratio (correlation coefficient: 0.24, *p* = 0.022). However, in the T2DM+HTH group, the TG level (correlation coefficient: 0.28, *p* = 0.012), TG/HDL ratio (correlation coefficient: 0.38, *p* = 0.001), and HOMA-IR were significantly associated with HDL cholesterol levels (correlation coefficient: −0.32, *p* = 0.003).

In the T2DM+HTH group (Table 2B), a decreasing HDL cholesterol level was significantly associated with HOMA-IR (correlation coefficient: −0.32, *p* = 0.005). We also observed an association between the FT4 and TG levels (correlation coefficient: 0.33, *p* = 0.018), between the TSH and HDL cholesterol levels (correlation coefficient: 0.30, *p* = 0.012), and between the TSH and LDL cholesterol levels (correlation coefficient: −0.26, *p* = 0.029).

### 3.4. Regression Analysis of Factors Related to Insulin Resistance in Patients with T2DM with Hyperthyroidism

To exclude the potential effects of abnormal liver functions, the serum glutamate–pyruvate transaminase (GPT) level was measured and found to be normal in all patients (mean: 26.7 U/L).

**Figure 2:**
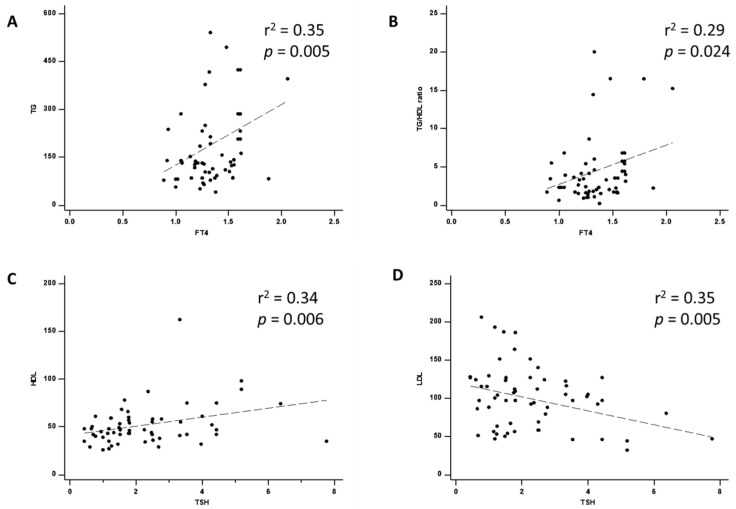
Regression analysis of the (**A**) TG level and (**B**) TG/HDL ratio with serum FT4 association and (**C**) HDL cholesterol and (**D**) LDL cholesterol with serum TSH association. TG: Triglyceride, HDL: High-density lipoprotein, LDL: Low-density lipoprotein, TSH: Thyroid-stimulating hormone, *p*: *p*-value; r^2^: r-squared.

The factors associated with insulin resistance were analyzed by univariate regression analysis. Linear regression showed that the TG level (*p* = 0.005, r^2^ = 0.35, Figure 2A) and TG/HDL ratio (*p* = 0.024, r^2^ = 0.29, Figure 2B) were significantly associated with the serum FT4 level in patients with T2DM with hyperthyroidism. Furthermore, HDL cholesterol (*p* = 0.006, r^2^ = 0.34, Figure 2C) and LDL cholesterol (*p*  = 0.005, r^2^ = 0.35, Figure 2D) were significantly associated with the TSH level in patients with T2DM with hyperthyroidism. Herein, we identified significant predictive abilities of the FT4 level with the TG level or TG/HDL ratio, and of the TSH level with the HDL cholesterol level or LDL cholesterol level.

### 3.5. Predictive Ability of FT4 and TSH for Insulin Resistance in Patients with T2DM

**Figure 3:**
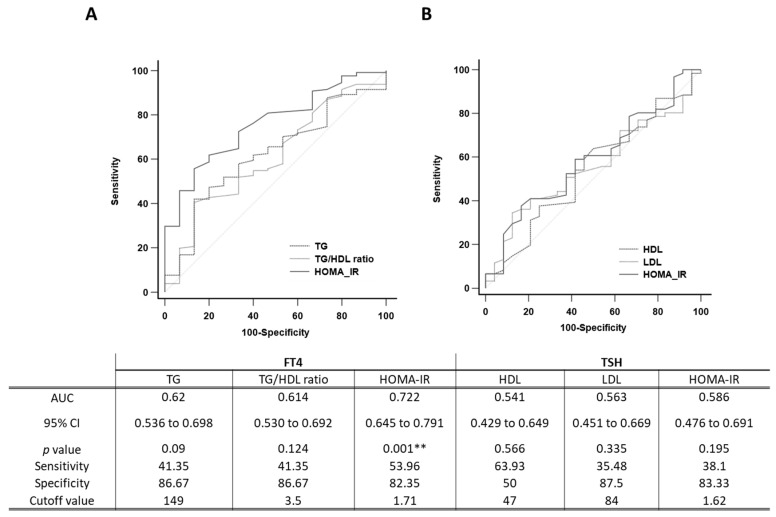
ROC curve base on the (**A**) TG level, TG/HDL ratio, and HOMA-IR with serum FT4 levels and (**B**) HDL cholesterol, LDL cholesterol, and HOMA-IR with serum TSH levels. TG, triglyceride; HDL, high-density lipoprotein; LDL, low-density lipoprotein; FT4; thyroxine, TSH, thyroid-stimulating hormone. ** *p* < 0.01.

To further examine the effects of FT4 and TSH variability in the total study population on insulin resistance, we plotted ROC curves and calculated AUC values to compare the predictive ability of the mean FT4 and TSH levels for insulin resistance (Figure 3). Among the different levels of serum FT4 in all patients with T2DM, the AUCs of the TG level, TG/HDL ratio, and HOMA-IR were 0.620 (95% CI: 0.536 to 0.698), 0.614 (95% CI: 0.530 to 0.692), and 0.722 (95% CI: 0.645 to 0.791), while the optimal cutoff values of the nomogram were 149, 3.5, and 1.71, respectively. The AUC value of HOMA-IR was higher than that of the TG level and TG/HDL ratio.

Among the different levels of serum TSH in patients with T2DM, the AUCs of HDL cholesterol, LDL cholesterol, and HOMA-IR were 0.541 (95% CI: 0.429 to 0.649), 0.563 (95% CI: 0.451 to 0.669), and 0.586 (95% CI: 0.476 to 0.691), while the optimal cutoff values of the nomogram were 47, 84, and 1.62, respectively.

The optimal cutoff values of the factors determined by ROC curve analyses for both FT4 and TSH are summarized in Figure 3.

## 4. Discussion

In patients with T2DM with hyperthyroidism, achieving glycemic control and avoiding insulin resistance remains a challenging task. Therefore, we used the clinical data and serum test results from patients to demonstrate that elevated thyroid hormone levels can indeed lead to increased insulin resistance in patients with T2DM, and that high levels of FT4 can indirectly increase the insulin resistance index by positively correlating with the TG levels. Additionally, we found that in patients with T2DM with hyperthyroidism, low TSH levels were positively correlated with HDL cholesterol levels and negatively correlated with LDL cholesterol levels, which indirectly increases the insulin resistance index.

The HOMA-IR index is widely used in many epidemiological studies and in clinical practice to estimate and evaluate insulin resistance in DM. Indeed, consistent with our findings, previous studies have reported the usefulness of the TG level and TG/HDL cholesterol ratio as predictors or markers of HOMA-IR [12]. However, the occurrence of hyperthyroidism may result in additional correlations.

Based on previous findings, elevated levels of FT4 can lead to a decrease in HDL cholesterol and an increase in TG and LDL cholesterol levels, causing increased insulin resistance in patients with hyperthyroidism [10,13]. This is similar to the results of our research; however, the situation is different in patients with T2DM. Our results revealed that the increase in HOMA-IR in patients with T2DM is related to an increase in the TG level and TG/HDL ratio. However, in the group of patients with T2DM combined with hyperthyroidism, HDL cholesterol levels were significantly negatively correlated, indicating that in these patients, HDL cholesterol levels appear to affect the severity of insulin resistance. These findings suggest that elevated FT4 and low TSH levels indirectly affect the levels of TG and HDL cholesterol, more severely affecting insulin resistance.

Previous research has shown that low TSH levels do cause an increase in TG and LDL cholesterol levels and a decrease in HDL cholesterol levels, [14] and that elevated FT4 levels do indeed increase TG and decrease HDL cholesterol levels in patients with hyperthyroidism [15]. These findings may be explained by the fact that FT4 increases intestinal glucose absorption, decreases insulin secretion, increases liver glucose production, and increases catecholamines, leading to insulin resistance [16,17,18].

The impact of hyperthyroidism on HDL may be multifaceted. In current studies, it has been reported that HDL may not only be associated with dyslipidemia, but also with microvascular complications of T2DM such as diabetic kidney injury [19]. Some studies have concluded that the binding of T4 to HDL is mediated by a specific interaction of the hormone with apolipoprotein A-I (apo A-I), the main protein of HDL [20]. Thus, hyperthyroidism is considered to be associated with decreased total and HDL cholesterol levels, total/HDL cholesterol ratio, and apo A-I levels [21]. Additionally, other studies have shown a decrease in HDL levels in patients with hyperthyroidism due to increased cholesteryl ester transfer protein-mediated transfer of cholesteryl esters from HDL to very-low-density lipoproteins and increased catabolism of HDL [14]. Further research in association between thyroid disease and diabetic micro-vascular complications may be necessary.

Despite these increases in understanding, the exact effects of insulin resistance, lipid metabolism, and thyroid function on each other are still debated. Insulin resistance may also occur in hypothyroidism, where there is a reduction in glucose-induced insulin secretion by β cells. However, in hyperthyroidism, insulin clearance is increased due to the response of β cells to glucose or increased catecholamines. Thus, insulin resistance may be present in both hypothyroidism and hyperthyroidism [11,17]. According to recent research, patients with hypothyroidism may also have severe insulin resistance or even reverse lipid metabolism, resulting in more severe lipid metabolism disorders [22,23]. However, regardless of the situation, insulin resistance seems to be an important variable affecting the relationship between T2DM and thyroid dysfunction.

A previous study has shown that insulin resistance and β-cell function are negatively correlated with TSH, possibly because higher serum TSH levels usually correspond to lower thyroid hormone levels through negative feedback mechanisms; indeed, as TSH increases, thyroid hormone decreases, and the insulin resistance effect weakens [24]. These observations suggest that insulin imbalance is closely related to thyroid dysfunction and abnormal phenomena caused by β-cell dysfunction.

Furthermore, in the present study, we found strong associations between FT4 levels and HOMA-IR in all study participants. Interestingly, this may indicate that in the T2DM population, regardless of the presence of hyperthyroidism, FT4 may be a predictor of insulin resistance; however, the reasons for insulin resistance vary among groups with normal and elevated thyroid function. Previous studies have also found that high FT4 levels unrelated to obesity are positively associated with HOMA-IR in healthy euthyroid subjects [25] and in postmenopausal obese women [26]. However, these factors influence both thyroid function and insulin resistance in participants, rather than having a directly causal relationship.

Our study has some limitations that warrant discussion. First, thyroid function was measured only via TSH and FT4. However, in clinical practice, as for hyperthyroidism, fT3 is not considered necessary as a surveillance biomarker; indeed, fT3 and T3 are tested only when a doctor suspects that a patient has associated hyperparathyroidism or a thyroid storm. Second, the number of cases (177 patients) enrolled in this study was relatively small, which may have led to sampling bias. Therefore, to increase the reliability of the reported data, we attempted to exclude many related chronic diseases and ensure homogeneity of the study population. Finally, to uphold the patient’s rights and follow the standard treatment protocol, our patients were included after receiving anti-thyroid medication. However, the effects of excessive FT4 levels could still be observed. In this study, we excluded cardiac disease or heart failure to reduce the bias caused by severe cardiac complications on serum thyroxine and blood lipids. However, based on the results of this study, the changes in serum markers in patients with thyroid disease and type 2 diabetes and heart comorbidities need further confirmation.

The lipid metabolism effects caused by endocrine and diabetes medications may be numerous, but we made every effort to minimize the differences between the two groups to align with real-world scenarios.

## 5. Conclusions

Our results suggest that elevated FT4 levels due to hyperthyroidism could alter the association with the lipid profile and insulin resistance in patients with T2DM. Our findings also suggest that among all the included patients with T2DM, irrespective of the presence of hyperthyroidism, FT4 levels are positively correlated with insulin resistance. Early detection of elevated FT4 levels due to hyperthyroidism in patients with T2DM may prevent the development of comorbidities and enhance disease management. Previous findings have suggested many complex physiological mechanisms underlying the endocrinology of insulin resistance and thyroid dysfunction. Therefore, further in-depth studies with a larger number of cases are warranted.

## Figures and Tables

**Table 1:** Clinical characteristics of the study population.

	Reference	HTH + T2DM	T2DM	*p*-Value
Age, mean (median)		53.1 (52)	53.2 (54)	0.121
Sex, male/female		60/19	69/29	0.799
Treatment duration, month (median)		72.6 (81.8)	75.8 (82.3)	0.572
Past history, n (%)				
Hypertension		12 (15)	19 (19)	0.587
Dyslipidemia		49 (62)	50 (50)	0.807
Cancer		0	0	
Stroke		0	0	
Heart failure		0	0	
NAFLD/NASH		0	0	
Antilipidemia drug, n (%)				
Atorvastatin		14 (17)	20 (20)	0.703
Rosuvastatin		35 (44)	30 (31)	0.879
Antidiabetic drug, n (%)				
Insulin glargine		7 (8)	8 (8)	0.807
Insulin degludec		4 (5)	8 (8)	0.165
Human insulin		2 (3)	5 (5)	0.446
GLP-1RA		2 (3)	2 (2)	0.176
Metformin		59 (75)	81(83)	0.051
Linagliptin		29 (24)	30 (31)	0.491
Vildagliptin		22(23)	30 (31)	0.099
Dapagliflozin		18 (20)	18 (17)	0.341
Empagliflozin+linagliptin		5 (6)	9 (9)	0.191
Saxagliptin+dapagliflozin		7 (10)	7 (7)	0.483
Antithyroid drug, n (%)				
Methimazole		70		
Propylthiouracil		1		
Laboratory results				
Glucose, mg/dL (SD)	70–100	153.1 (58.3)	134 (40.4)	0.024 *
Insulin, µIU/µL (SD)	1.9–23.0	14.1 (11.9)	3.4 (2.1)	0.005 **
HOMA-IR, ratio (SD)	<1.4	3.8 (2.4)	1.7 (0.1)	0.001 **
HBA1C, % (SD)	<5.5	7.4 (1.7)	7.6 (2.1)	0.884
LDH, IU/L (SD)	98–192	231.3 (82.7)	200.3 (58.8)	0.397
TG, mg/dL (SD)	<150	184.1 (122.9)	147.3 (92.0)	0.047 *
HDL, mg/dL (SD)	>40	54.1 (24.0)	45.8 (12.0)	0.019 *
LDL, mg/dL (SD)	<130	100.2 (40.3)	94.5 (44.6)	0.183
TG/HDL, ratio (SD)		4.3 (4.0)	4.2 (5.2)	0.927
TC, mg/dL (SD)	<200	189.1 (51.6)	172.9 (55.3)	0.070
FT4, ng/dL (SD)	0.7–1.4	1.42 (0.3)	1.22 (0.2)	0.001 **
TSH, µIU/dL (SD)	0.5–5.0	2.6 (1.8)	2.9 (1.6)	0.522

T2DM: Type 2 diabetes mellitus, HTH: Hyperthyroidism, NAFLD/NASH: Non-alcoholic fatty liver disease/nonalcoholic steatohepatitis, GLP-1RA: Glucagon-like peptide-1 receptor agonist, HOMA-IR: Homeostasis model assessment-insulin resistance index, HbA1C: Glycated hemoglobin A1c, LDH: Lactate dehydrogenase, TG: Triglyceride, HDL: High-density lipoprotein, LDL: Low-density lipoprotein, TC: Total cholesterol, FT4: Thyroxine, TSH: Thyroid-stimulating hormone. * *p* < 0.05, ** *p* < 0.01.

**Table 2:** Correlation of the HOMA-IR index with other related hematological factors in (**A**) the T2DM group and (**B**) the T2DM + HTH group.

**(A)**										
	HOMA-IR	HBA1C	LDH	TG	HDL	LDL	TG/HDL	TC	FT4	TSH
HOMA-IR	1									
HBA1C	−0.05	1								
LDH	0.10	−0.16	1							
TG	0.25	0.14	−0.09	1						
HDL	−0.18	−0.29	0.21	−0.45	1					
LDL	0.04	0.09	0.11	0.14	0.07	1				
TG/HDL	0.24	0.22	−0.16	0.94	–0.69	0.08	1			
TC	0.10	0.03	0.08	0.37	0.13	0.87	0.23	1		
FT4	0.11	0.01	−0.12	0.14	−0.04	0.14	0.10	0.21	1	
TSH	−0.11	0.03	0.06	0.14	−0.07	−0.01	0.15	−0.05	−0.18	1
**(B)**										
	**HOMA-IR**	**HbA1C**	**LDH**	**TG**	**HDL**	**LDL**	**TG/HDL**	**TC**	**FT4**	**TSH**
HOMA-IR	1									
HbA1C	0.07	1								
LDH	−0.11	0.32	1							
TG	0.28	−0.02	−0.10	1						
HDL	−0.32	−0.01	0.10	−0.38	1					
LDL	0.06	−0.13	0.12	0.15	−0.18	1				
TG/HDL	0.38	−0.03	−0.02	0.92	−0.66	0.19	1			
TC	0.22	−0.17	0.13	0.49	−0.01	0.78	0.42	1		
FT4	0.01	0.04	−0.23	0.33	0.12	0.14	0.24	0.24	1	
TSH	−0.14	−0.06	0.01	–0.07	0.30	–0.26	−0.18	−0.13	0.11	1

The deep gray color labels indicate *p* < 0.01, and the light gray color labels indicate *p* < 0.05. HOMA-IR: Homeostasis model assessment-insulin resistance index, HbA1C: Glycated hemoglobin A1c, LDH: Lactate dehydrogenase, TG: Triglyceride, HDL: High-density lipoprotein, LDL: Low-density lipoprotein, TC: Total cholesterol, FT4: Thyroxine, TSH: Thyroid-stimulating hormone.

## Data Availability

The datasets used and/or analyzed during the current study are available from the corresponding author upon reasonable request.
